# Contextualizing Security Innovation: Responsible Research and Innovation at the Smart Border?

**DOI:** 10.1007/s11948-021-00292-y

**Published:** 2021-02-18

**Authors:** Nina Klimburg-Witjes, Frederik C. Huettenrauch

**Affiliations:** 1 Department of Science and Technology Studies, University of Vienna, Universitätsstrasse 7, 1010 Vienna, Austria; 2 School of Management, Technical University Munich, Arcisstrasse 21, 80333 Munich, Germany

**Keywords:** Responsible research and innovation (RRI), Security, Innovation, Smart borders, Science and technology studies (STS)

## Abstract

Current European innovation and security policies are increasingly channeled into efforts to address the assumed challenges that threaten European societies. A field in which this has become particularly salient is digitized EU border management. Here, the framework of responsible research and innovation (RRI) has recently been used to point to the alleged sensitivity of political actors towards the contingent dimensions of emerging security technologies. RRI, in general, is concerned with societal needs and the engagement and inclusion of various stakeholder groups in the research and innovation processes, aiming to anticipate undesired consequences of and identifying socially acceptable alternatives for emerging technologies. However, RRI has also been criticized as an industry-driven attempt to gain societal legitimacy for new technologies. In this article, we argue that while RRI evokes a space where different actors enter co-creative dialogues, it lays bare the specific challenges of governing security innovation in socially responsible ways. Empirically, we draw on the case study of BODEGA, the first EU funded research project to apply the RRI framework to the field of border security. We show how stakeholders involved in the project represent their work in relation to RRI and the resulting benefits and challenges they face. The paper argues that applying the framework to the field of (border) security lays bare its limitations, namely that RRI itself embodies a political agenda, conceals alternative experiences by those on whom security is enacted upon and that its key propositions of openness and transparency are hardly met in practice due to confidentiality agreements. Our hope is to contribute to work on RRI and emerging debates about how the concept can (or cannot) be contextualized for the field of security—a field that might be more in need than any other to consider the ethical dimension of its activities.

## Introduction

Current European innovation policies are increasingly channeled into efforts to address the assumed challenges that threaten European societies. Innovation is thereby often presented as “the only solution” for problems as complex and diverse such as climate change, terrorism, or food security (EC 2010: 6). A field in which calls for innovation have become particularly salient is that of digitized EU border management. In 2013, the European Commission launched the Smart Borders Package that introduced automated border control technologies, Entry/Exit systems to track traveler information (Le Guellec et al. 2018) and biometrics-based authentication systems such as iris scans and fingerprints (Weissenfeld et al. 2018). As Trauttmansdorff (2017) has argued, the Smart Borders Package demonstrates particularly well the essential role of digital technologies within the EU’s ‘Integrated Border Management’ strategy, interlinking cross-border crime, terrorism and irregular migration and establishing an ‘immigration/security nexus’ (Van Munster 2009: 46). The strategy emphasizes the need for an open and secure Europe to be achieved by technological innovation in the form of an ‘extensive toolbox for collecting, processing and sharing information between national authorities and other players’ (CEU 2010: C115/18). While the Smart Borders Package was officially heralded as bringing about the ‘border of the future’ (EU-LISA 2015, in: Sontowski 2017: 2), and to ‘facilitate, speed-up and reinforce the border check procedures at EU’s external borders’ (EC 2013: 2) it spurred controversies as well: Critical assessments saw digitized borders as another tool of ‘mass dataveillance’ (Jeandesboz 2016, 305), enacting an economic sorting mechanism in the name of security’ (Leese 2016: 415, cf. Kloppenburg and Van der Ploeg 2020) with security technologies such as biometrics being particularly prone to reproduce and reinforce power imbalances and social injustice (Leese et al. 2019: 60).

Largely driven by IT specialists instead of border guards, Bigo (2014: 217) has argued that the digitization of borders follows a rationale that “technology solves the tension among an open economy, freedom of circulation of travelers and control, via dataveillance”. What is more, the emphasis on “smartness” by EU policymakers has been criticized as closely entangled with industry-driven attempts to obtain societal acceptance for novel technologies (Bigo et al. 2012; Scherrer et al. 2011; Wright et al. 2010). Indeed, gaining acceptance and governing emerging technologies and innovation in a socially desirable way is a task that is difficult to accomplish (Winickoff and Pfotenhauer 2018), even more so when it comes to security innovation. Here, the term ‘responsible’ is frequently evoked to point to the alleged sensitivity of political actors towards the negative and restrictive dimensions of security technologies (cf. Burgess et al. 2018).

The framework of Responsible Research and Innovation (RRI) was specifically developed to address the social and political challenges brought by new technologies (Morris et al. 2011) in all kinds of political, social and economic realms. It aims at bringing about a more sustainable transition of social and technical arrangements (Geels 2010), and foster a stronger engagement between science-driven innovation and society (Delgado and Åm 2018; Wynne 2001; Smith and Tidwell 2016). In the field of border security, too, policymakers, industry stakeholders and technical experts have turned to RRI as a ‘panacea’, a universal remedy, “that carries the promise of curing socioeconomic ailments almost irrespective of what these ailments are or how they have arisen” (Pfotenhauer and Jasanoff 2017: 784). Despite its salience in both EU policy-making and social science accounts, the application of RRI and its contextualization for the field of security innovations have received little attention to far; a lacuna given the relevance of forms of responsible governance at the nexus of research, innovation and security.

In what follows, we will explore how RRI, including its normative assumptions of increasing the social benefits from technological innovation through societal participation, comes to matter in the planning, testing and implementation of smart borders. To do so, we draw on the case study of an EU-funded research project, BODEGA, which is the first EU initiative to apply the RRI framework in the field of security. Drawing on ten interviews with members of the BODEGA project between 2018 and 2019, we trace the underlying assumptions, challenges, and tensions with applying RRI principles to the process of digitizing the EU’s external borders. The project specifically focusses on the introduction of Automated Border Control systems (“Smart Borders”) and eGates at the EU Schengen border. eGates are expected to play a crucial part in dealing with increasing traveler flows that manual border checks are said to be unable to cope with. As a result, Smart Borders introduce a shift from local physical borders operated by border guards to computer checks (cf. Bigo et al. 2012: 36). The changing role of the border guards is the main concern of the BODEGA project that aims to apply RRI principles to all steps of the research process to understand and facilitate this transformation.

The paper unfolds as follows: First, we provide a review of the current body of work on the RRI framework. Second, the empirical part examines how stakeholders involved in the project represent their work in relation to RRI and the resulting benefits and challenges they face in the process of applying RRI to the field of automated border control. In the last section, we argue that while RRI evokes a space where researchers and engineers can enter into dialogue and co-create practices with end-users, it lays bare the specific challenges of governing security innovation in socially responsible ways.

The overall aim of this article is two-fold: First, to contribute to the ongoing work and discussions on RRI by offering a case study of security innovation and thus spur a debate on how the concept can (or cannot) be contextualized for the field of security. Second, to add to both academic and policy debates on EU Smart Borders concerned with privacy and surveillance issues and migrant rights by introducing RRI as a conceptual lens for future work to explore how questions of anticipation, reflexivity, inclusion and responsiveness can be addressed and debated in the field of EU digital border management.

## Theoretical Background

### Responsible Research and Innovation

The concept of RRI has gained in importance in European policy debates, in particular in the course of the ‘Horizon 2020’ framework (see EC 2019). The framework emerged from research activities around ethical, legal, and social aspects (ELSA), technology assessment (see Schot and Rip 1997) or anticipatory governance (Karinen and Guston 2009) and today comprises several strands. A frequently used definition is that RRI “is a transparent, interactive process by which societal actors and innovators become mutually responsive to each other with a view on the (ethical) acceptability, sustainability and societal desirability of the innovation process and its marketable products (in order to allow a proper embedding of scientific and technological advances in our society)” (Von Schomberg 2011: 9). Stilgoe et al. propose a broader, more open definition where “[r]esponsible innovation means taking care of the future through collective stewardship of science and innovation in the present”. (2013: 1570). For them, RRI is characterized by four dimensions that derive from science and technology-related public discussions: anticipation, reflexivity, inclusion, and responsiveness.

*Anticipation* is a frequently recurring theme in the governance of science, (emerging) technologies and innovation and their socio-political implications (e.g., Jasanoff 2016, 2003a, b; Stirling 2010). This conceptual dimension requires scientists, researchers and innovators to consider various scenarios that might occur in the course of their research and innovation activities and to reflect on the dynamics that are shaping the design of their work (Jacob 2013).

*Reflexivity* urges those in charge of the innovation process “to blur the boundary between their role responsibilities and wider, moral responsibilities” (Stilgoe et al. 2013: 1571, cf. Schuurbiers 2011). The demand for *inclusion* is rooted in concerns about expert-driven top-down policy activities (Felt et al. 2007; Burget et al. 2017) and calls for public participation in science and innovation activities for identifying socially desirable outcomes (cf. Von Schomberg 2007; Owen et al. 2012; Stahl 2013; cf. Macnaghten and Chilvers 2014).

Lastly, *Responsiveness* is supposed to change the trajectory of the development and avoid undesired consequences (Jacob 2013), considered as “an encompassing yet substantially neglected dimension of responsibility” (Pellizzoni 2004: 557) and linked to questions of ethics, values, transparency, norms, accessibility or risks during the research and innovation process (Forsberg et al. 2015; Frewer et al. 2014; Levidow and Neubauer 2014). These four dimensions are mutually interrelated, creating synergies but also tensions in the process to ensure a responsive character of responsible innovation (Stilgoe et al. 2013).

In sum, RRI initiatives are concerned with societal needs or challenges and the engagement and inclusion of various stakeholder groups and aim at anticipating undesired consequences and identifying socially acceptable alternatives (Wickson and Forsberg 2015: 1164). To operationalize the framework, the EC considers six key elements—engagement, ethics, gender equality, open access, science education and governance—as fundamental fields of action for all RRI initiatives (Table 1).

**Table 1:** The six keys of the RRI framework defined by the European Commission

No	Key element	Description
1	Engagement	Promote research by including societal actors
2	Ethics	Preserve universal values and enhance society’s wellbeing
3	Gender equality	Avoid gender imbalances and ensure a holistic, gender-neutral view
4	Science education	Improve educational processes and provide knowledge to enhance science and innovation
5	Open access	Share scientific knowledge among researchers and citizens and improve transparency
6	Governance	Provide infrastructure to steer research and innovation in a socially desirable way

### Critique of the RRI Framework

Recently, scholars from different fields have criticized the ambiguity of the term RRI as well as the framework’s interpretative flexibility (e.g. Bensaude Vincent 2014; Oftedal 2014; Owen et al. 2012; Ribeiro et al. 2017). The fact that there is neither a commonly agreed definition nor a clear way how to implement RRI is a recurring criticism: The framework is considered “overly vague” (Wickson and Carew 2014: 256) missing guidelines and measurement criteria. From an industry-oriented perspective, Blok and Lemmens (2015) also question the practical applicability of the framework and call for a more thorough inquiry of the concept, criticizing the narrow focus on research and innovation activities in academic environments. This, they state, is problematic, since most innovations take place in the private sector, making current proposals for RRI highly questionable for business purposes (cf. Fisher and Rip 2013; Blok et al. 2015). Felt (2018, p. 112) has argued that RRI is often used “as a mechanism to grease the wheels of technological progress” instead of embedding profound values in the research process, while Åm (2019) calls for creating sufficient conditions for novel, and more responsible practices instead of solely encouraging scientists to adopt responsible behaviors.

Yet, accounts that contextualize the application of RRI for the field of security and offer a critical exploration of the potential problems it might cause when applied to security research are scarce. A valuable exception being the works by Burgess et al. (2019) and Rychnovska (2016) respectively. The former problematizes the implications of new technologies for the governance of security and more responsible forms of innovation, however, without an explicit focus on RRI. Drawing on the case of dual-use knowledge^1^ in the life sciences, Rychnovska argues that the converging political rationalities and governmental techniques of responsible science and security risk management understood as an ‘ethicalization’ of security, affect the politicization of security expertise, the prospects of resistance and the democratic accountability of science. When concerns about moral and ethical responsibilities of science become a matter of security, she asks, to whom then will science and innovation be responsible? (2016: 323). With this article, we aim to contribute to the emerging body of research that aims at contextualizing RRI for the field of security through an empirical investigation of the challenges and benefits encountered by those who applied RRI principles to the field of (researching) border security and automated (“smart”) border control systems.

## Material and Methods

In this paper, we draw on the case study of the BODEGA,^2^ the first EU-funded research project to apply RRI in the context of border security. It aims at developing “future border checks with human factors expertise in order to enhance efficiency, border security and traveler satisfaction” (BODEGA 2018) by applying the RRI framework to the research process. The project was funded through Horizon 2020^3^ in the funding scheme ‘Secure societies—Protecting freedom and security of Europe and its citizens’ (Cordis 2017) and was conducted between 2015 and 2018. BODEGA is organized as a consortium of 16 partners from seven European countries, spanning from universities, industry stakeholders, national research institutes and digital agencies. A key focus of the project is to understand the implications of smart border control systems and biometrics-based self-service systems for traveler processing and the working conditions of border guards at Schengen border crossing points with a special focus on airports (BODEGA 2018) as those sites where most of the economic benefit is generated by travel and smooth operations are crucial, e.g., by avoiding long queues. By applying RRI, the participating organizations aim to ensure that the technology development work is participatory and considers foresight-related issues and ethical and societal aspects. (Pearson et al. 2016; Cordis 2017). As one of the central aims of the project is the engagement of all stakeholders affected “by the development and automation of border checks and border control processes” (Toivonen et al. 2016: 7), Table 2 gives an overview of the stakeholder groups of the BODEGA project.

**Table 2:** Relevant stakeholder groups in the BODEGA project

No	Stakeholder group	Description
1	National border control agencies/other operational stakeholders	Primary stakeholder group in the border context. Divided into national border control agencies and other operational stakeholders who oversee border management and control tasks. Considered as end users from BODEGA’s perspective
2	Technical stakeholders	Includes technology providers, technology related European research projects in the field of border control, standardization organizations, associations in the field of security, biometrics, or border control as well as private and public research institutes
3	Policy stakeholders	Main actors are the EU Parliament, the EU Council and the EU Commission including the Directorate-General for Migration and Home Affairs as well as national policymakers from EU member states, who affect the border space at all different levels
4	Societal stakeholders	Includes a broad range of actors from society, from travelers to research and academia to civil society, including the media, experts for human rights, data privacy or ethics, NGOs and employer associations

The core of the empirical material for this paper are ten semi-structured expert interviews with stakeholders from the BODEGA consortium. The interviews were conducted between June and September 2018, either via Skype or phone. While three interviewees were researchers with in-depth knowledge about RRI from previous EU-funded projects, seven interviewees, representing national border authorities and industry were not familiar with RRI. All interviews were anonymized, and quoted by agreement. The interviews were transcribed and analyzed using a constructivist grounded theory approach (Charmaz and Belgrave 2012; Ryan and Bernard 2000) that draws the researcher’s attention to the social interactions and experiences of people with a certain phenomenon, in this case, RRI in the context of security innovation (Ryan and Bernard 2000: 782; Charmaz 2006: 130–131; Saunders et al. 2009). For the analysis, all interviews have been coded using the qualitative data analysis software ATLAS.ti. We conducted a close line-by-line coding, i.e., each line of the interview transcript was labelled with a code (such as “challenge”, “responsibility”).

The initial codes have been used to identify broader categories such as “confidentiality” and “participation”. In a second step, these were annotated and assigned more specific codes that were representative of the practices and relations in that category. We could then utilize these codes to identify patterns, specifying the exchanges and interactions between the different groups of actors involved, their respective experiences with RRI, and their sense-making practices when applying the framework to the project.

## Empirical Analysis

### Background: Transforming the Role of the Border Guard

One of the key propositions of the project under study was that with the increasing use of automated systems (e.g., eGates) at the Schengen borders, border guards would have more capacities to focus on the identification of high-risk travelers. Simultaneously, the introduction of eGates was expected to significantly transform the role of border guards as they introduce a physical distance between the border guard and the traveler. Instead of interacting with travelers directly, border guards will work rather remotely to monitor the border control processes. One of the BODEGA project's key goals was to “enhance the border control efficiency without side effects to the end-users” (Papillault et al. 2016: 6). Thus, the project consortium anticipated potential issues and challenges for both border guards and travelers to occur with the digitization of the border space. To reflect during the process which actors might be affected in what ways by the introduction of smart borders, the project applied the RRI principles “a must for this kind of project” (IP8) as one interviewee stated, to bring all the different stakeholder together.

### Understanding the Implications of Emerging Technologies

To understand the implications of the transformation of the border space, stakeholders of the BODEGA project stated that they applied a holistic approach and considered border controls as a complex social phenomenon including “identity management, security and law enforcement, surveillance, customs, protection of vulnerable people, rescue, etc.” (Nikolova and Goujon 2016: 1). The project used the six keys from the EU RRI framework (see p. 3) in a flexible way, only loosely following the definitions as to adapt it to the specific context of border control (IP 6, cf. Gianni and Ikonen 2016: 1). Considering the broader socio-technical context of the border space and its expected transformation as at least controversial, many project members emphasized, in particular, the need for reflexivity in the project, in the form of an “open room for alternative possibilities for action and change” (Nikolova and Goujon 2016: 1). However, this open room was understood as confined to the scope of the project and the respective actions and decisions of the stakeholders involved and did not refer to broader questions of EU migration and security policy. It was aimed at understanding the specific work tasks of the participating organizations and find ways to collectively attend to questions of responsibility during the research and development process. One industry actor stated that in the field of security innovation and technology development “[s]ometimes the need to have a technology comes first and sometimes technology is implemented before considering and understanding its full implications” (IP 1). According to this interviewee, RRI allowed team members to jointly reflect on the possible implications of novel technologies in a way that would not have been possible otherwise. This became especially evident through the project’s engagement with the perspectives of travelers when crossing borders and using eGates, and their “expectations and even their fears about technology” (IP 6).

An industry actor remembers that one of the first things they realized during the research process was the reluctance of many travelers “to use new technologies, basically because they do not trust the usage of biometrics or the usage of their personal data for border management” (IP 8). For the industry actors, being “nudged by RRI” (IP6) to reflect more thoroughly on the technologies they co-developed helped anticipate undesired consequences such as gender or racial biases during the control process more proactively. However, the project addressed these issues by mainly focusing on the border guards: For instance, researchers mentioned that they learned from a female border guard that the safety vest she had to wear was not well fitted to her body shape as it was originally designed for men, thus making her uncomfortable in her daily work. The researchers stated that without RRI, that would not have attended to concerns such as this one. Engaging with end users—travelers and border guards—was considered “the appropriate way of dealing with contingent issues” (Gianni and Ikonen 2018, p. 1) to discuss the different perceptions of automated border controls. Here, much emphasis was put on the mutual learning between the project members and the users of automated border gates, as the following statement indicates: “It’s not only the researcher or the company who gets insights from the end-user. But also, the end user is learning something when you interact with them”. (IP 6).

What was left absent in the accounts of interview partners, however, was the perspective of those who were denied access at the border or even before that, to the airport. Rather, mutual learning was appreciated as a way to add an end-user perspective to the development process to increase efficiency while educating the user was seen as a way “to gain acceptance for the new technologies” (IP1) such as eGates to be implemented and the technical specificities of their usage as well as concerns about data protection. While such a focus on mutual learning is compliant with the key assumptions of RRI, it also reflects some of the basic tenants of the public awareness of the science movement: The aim to foster attitudes and behaviors more favorable to science and novel technology among citizens through education and to turn to co-creation as a remedy for dissent and conflict around science and technology. In that sense, educating “reluctant” travelers “to be less afraid” of the digitized border then already implies assumptions about a desirable social, political, and economic order (Jasanoff 2016, 2015). In other words, by searching for solutions to potential challenges from automated border controls, stakeholders turned to RRI and by that already mobilized, performed, and reconfigured what would be desirable socio-technical configurations (Wentland 2016)—a smooth and speedy process of (self-)operating the smart border while gaining acceptance by travelers and border guards for the introduced transformations.

### Creating Spaces for Dialogue?

When it comes to the project’s internal processes and practices, most interviewees stated that it allowed them to better account for the implications of their work. In particular, the RRI indicators were seen by most interviewees as valuable for organizing the joint research process of the project, in particular, to create and sustain shared spaces for the different disciplines and interest groups involved to discuss what they saw as responsible development of automated border controls. (Gianni and Ikonen 2016).

Interviewees mentioned that those meetings and workshops specifically dedicated to the application of RRI allowed to raise certain ethical issues that had otherwise been silenced, as this statement shows: “These stakeholder meetings were very helpful because often public debates around societal issues can be quite nasty and people have agendas”. (IP3). This interlocutor referred to previous experiences from projects related to the development and application of security technologies that would often get “attacked by people who don’t understand [what he saw as the necessities of security governance, such as collaborating with border authorities and the police] or because of the political or social aspects of the project”. (IP3) Without explicitly mention what these aspects have been in the past, the interviewee differentiated between the “outside”, e.g., the public, seen as rather critical of security innovations and the “inside”, those collective of researchers, industry stakeholders and policy makers of the project with whom “you can start talking about these issues in a less threatening environment and actually discuss things” (IP 3).

This quote implies that the application of RRI principles within the project context creates a safe environment for those involved to meet with various other stakeholders and openly discuss their concerns about security research and innovation in the field of smart borders “away from political agendas” (IP 2). Within this space, the normative orientation of the RRI framework such as ethics, inclusivity and gender equality has helped “to keep together different drivers and apparently clashing logics under one main umbrella” (Gianni and Ikonen 2016: 1). Yet, inclusivity referred rather to the project consortium and not to broader public debates surrounding smart borders and EU migration politics in general.

While it was challenging for some of the industry actors in particular to balance ethical (e.g. data protection *for* travelers and transparency of the R&D process) and security considerations (data protection *from* the travelers and industrial secrecy), they emphasized the positive effect of collaborating with RRI experts who spurred a discussion of otherwise neglected debates and concerns.

For the industry actors, it was especially the collaborative setting created by the project which they found most beneficial: Those aspects and concerns that usually do not “have a place” in the R&D process were acknowledged in the project in which “RRI worked like an umbrella that encompasses everything”. (IP 8). All interviewees emphasized the need to address ethical values such as fairness and non-discrimination in the development of border management technologies. In this project, the RRI thus worked as an integrative governance framework that allowed to bring together the different perceptions, values, and expectations among the stakeholders regarding automated border control technologies and their sociopolitical implications. However, this did not always mean to open up the process to broader societal debates. Rather, keeping the “publics” out—those who either would not understand or politicize the issue at stake—was what made those within the project feel that they were inhabiting a safe space with the help of RRI.

### Challenges and Struggles with the Concept

Interviewees stated that they also experienced difficulties and challenging moments during the project when they tried to apply RRI. A recurring theme was the complexity of the framework, which was often perceived as a burden due to the additional work it requires. Both RRI and industry actors mentioned that the framework was too rigid and not well-suited for their everyday work practices and would impose “more bureaucracy, more paperwork, and more exercises” which in the end would not “really mean anything” (IP 3). In turn, RRI actors, those with previous experiences and responsible for bringing the framework to the project, saw their collaborators from engineering and industry as being too narrowly focused on aspects of technology development and little aware of, or interested in, the socio-political implications of their work. For those coming from the industry, the lack of indicators to measure the success of the framework was a constant struggle: they complained that RRI would be all about talking and less about clear guidelines to be implemented. These concerns were acknowledged by one of the RRI actors, who admitted that with RRI, “you can have a nice stakeholder session where you talk about things, but it doesn’t generate any parameters or indicators. Maybe that is difficult for people from a business context or a hard sciences context to cope with”. (IP 3).

This quote nicely illustrates the conflicting logics between RRI actors and industry actors in the project. RRI actors were hesitant to suggest metrics for measuring the impact of RRI as they perceived the framework rather as a long-term effort to create a space for open discussions. Yet, that logic clashed with the industry actors’ desire to follow clear guidelines and measure the short-term results of their efforts. Also, while RRI actors expressed the idea of introducing legal regulations or certifications for projects that adopt RRI more broadly, they simultaneously emphasized that applying RRI should always be a voluntary action. Rather than enforcing or imposing RRI on the other stakeholder groups, they saw it as their responsibility to make engineers and industry actors “understand that acquiring knowledge from end-users or other people is always something that makes you improve the process” (IP 2), thus in a way turning the Public Understanding of Science perspective the other way around: This time, not users have to be educated but were seen as a resource to enhance the innovation process.

Those with an RRI background conceived of ethics and technology as complementary to each other, suggesting that industry and engineering stakeholders would eventually need “to gain legitimacy for their technologies, products and processes in the long run” (IP 2). Still, convincing industry stakeholders and those from the natural sciences and engineering of what they saw as the values and benefits of RRI—citizen participation, co-creation and transparency of the innovation process, was sometimes also perceived as a time-consuming, cumbersome process by RRI experts. Summing up the experiences he and colleagues made during the project, one researcher with an RRI background stated that the framework was often seen to be mainly “promoted by people who are very theoretical and have a background in a kind of high-level philosophy and political theory” (IP 3). This quote shows that the RRI framework did not appeal to all stakeholders in the same way, since those from outside academic and/or with a more technical background did not see their working practices and valuation logics met, but rather conceived of RRI “as something nice if you have time for this”. (IP2).

A solution often proposed by RRI actors was to strengthen forms of interdisciplinary communication about the benefits of RRI beyond a rather exclusive community of already RRI inclined actors and experts. This was rather lacking, as interviewees with a background in engineering stated: “Because in RRI, they are talking about engaging people, talking with people, bottom-up approach, but if you go to a conference about RRI or an RRI workshop: who are you going to find there? Only RRI people”. (IP 2). Statements like this imply that the overall RRI discourse would need to improve in terms of its engagement with other actors from various fields to better account for the different demands, the perceptions on what responsibility in research and innovation processes should entail and how the process of applying (and contextualizing RRI for security fields) should be designed and structured. On the other hand, the interviewed RRI actors felt that technical and industry actors had a very restricted and limited view on technology development and its social and ethical implications. They feared that those stakeholders would only pursue those aspects and requirements of the framework convenient for them in their specific (work) context while neglecting the actual meaning of the framework and the work required to apply the principles to the contested field of (border) security. In the following section, we will attend to the specific challenges that the adoption of RRI to the field of security brings about.

### Adapting the Framework to the Needs of the Field: When Security Considerations and RRI Clash

The contextualization of the RRI framework and the question of how to adapt it to the specific requirements for projects on security-sensitive issues was a main concern of the project. This was especially the case when interviewees saw the foundational ideas of RRI and security considerations as standing in conflict with each other. As one interviewee stated, “If you come with a framework and tell people you have to do this and that and you don’t adapt it to the reality in the field, then it is useless. You need to understand people’s constraints”. (IP 5) The security-sensitive aspects of border security were a reality, as industry stakeholders stated, which the RRI framework was not able to address or capture. Several stakeholders referred to the difficulties in assuring an open process of security research and innovation as well as navigating the fine line between openness and practices of secrecy (e.g., the confidentiality of most of the project’s findings as required by its funding institution, the EC). For some, it was rather the interaction with the travelers that they found challenging, for instance when they had to explain why the project team cooperates with authorities like national police or FRONTEX, the EU border management agency “in order to keep the border secure (IP 6)”. For others, it was the security imperative of the project in general that was considered problematic.

The normative values of RRI are, amongst others, openness and inclusivity of the research process. Yet, in a project concerned with border security, it was precisely these values that were posing a problem. The following statement by an engineer of the project illustrates how the logics of RRI and security innovation can stand in conflict to each other when it comes to sharing information and enhancing mutual learning between security researchers and their subjects of study: “People know what you do and how you operate. This is also information for the bad guys. I understand the need for transparency, but at some point, if you want to arrest people who misbehaved, you need to ensure that not everything is known by everyone” (IP 5). For many interviewees, but in particular those with an academic background and employed at research institutions, it was important that to make sure only the “right people” got access—and not the “wrong” ones, when they were presenting their findings. This is when they found it hard to reconcile the different logics of RRI—e.g. making the research process publicly accessible as well as security innovation—with that of operational security, e.g. preventing potential criminals finding out exactly what border guards are looking for.

However, the interesting point here is not so much that some security technologies and their application often call for a certain level of confidentiality. Rather, it is how participants of a project dedicated to the application of all RRI principles invoke the notions of the “wrong people” or the “bad guys” who should not have access to the findings, while regretting to not be able to better inform the “right people” (e.g., travelers eligible to cross Schengen borders) about the project and its outcomes. These statements allude to the challenges RRI poses for security-related projects, such as allowing for open (access) knowledge while simultaneously making sure that security considerations are met.

A similar tension became visible about the questions of open access and industrial security innovations, although for different reasons. Here, interview partners from universities stated that they were facing several challenges when dealing with private companies that were “quite reluctant to sharing information”. (IP 8) This aspect resonates in other interviews, where company research was seen as “secretive” and “protective”, leading to “conflicts between this open access requirements, the open data requirements from Horizon 2020 funding and the research and design priorities of companies, who want to protect their innovations and their strategies”. (IP 3) This seems to be an inherent and unavoidable conflict between the framework itself and the security sector, that is, when industry innovation and security considerations merge, there seems to be not much room for the values proposed by the RRI framework. For the BODEGA research project and other research projects dedicated to applying RRI to the field of security, the different logics of openness (RRI) and secrecy thus seem hard to reconcile.

## Discussion and Conclusion

In this article, we explored how the framework of RRI is applied in an EU funded research project dedicated to the planning, testing and implementation of smart borders. Stakeholders in this project departed from an understanding that automated border controls will increase the digitization of the border space and by that, fundamentally affect the role of the border guard and the experiences of travelers. By applying the framework of RRI, the project consortium aimed to better understand the implications of these smart border control and biometrics-based self-service systems for traveler processing, their effects on the work of border guards and to identify possible ways to gain broader social acceptance of this transformation. Searching for solutions to potential challenges from automated border controls, stakeholders turned to RRI as a means to align technology to the needs of society, with the normative aim that technology should be for the benefit of society. Indeed, the RRI dimensions of reflexivity and engagement have substantially influenced the research process itself: they urged involved stakeholders to reflect upon their work whilst considering the perceptions of automated border controls by external stakeholders and end-users (border guards and travelers) through multiple forms of engagements.

Publicly funded research projects like the case study of this paper can therefore be understood as a test-bed for new modes of collaborative technology governance (cf. Engels et al. 2019): Within the project, researchers investigating RRI were able to test the framework and explore the tensions it creates under real-world conditions. Commercial industry actors and engineering stakeholders were able to experiment with the framework beyond the constraints of regulated markets and without interfering with their R&D activities (Engels and Rogge 2018). At the same time, publicly funded projects such as BODEGA provide spaces for the European Union to further promote and disseminate the RRI framework and specific aspects of its policy agenda in the field of smart borders.

However, while the application of the RRI framework has indeed led to novel forms of interaction and engagement among the project stakeholders, RRI cannot simply be considered an instrument that allows researchers and civil society to make their voice heard. Rather, it created what interview partners described as “safe space” for the different actors involved (e.g., border security, academia, engineering and industry) to discuss security innovation for border management “away from political agendas”.

While the project was not concerned with questions of EU migration policies and migrants’ rights in general, the argument of the safe space that RRI creates for those inside of the project still raises important questions that have been rather absent from the literature on RRI so far. First, the diversity of experiences with smart borders: Potential societal benefits of such technologies and their political implementation largely depend on which society one belongs to, in other word, on which side of the smart border one is standing. How could RRI and its values of inclusion and engagement then be thought of and applied to the field of border technologies that are per definition exclusionary (Bigo 2014)? How, if at all, can a project applying RRI to this context include migrant’s expectations and experiences, in particular of those being rejected by technology that has been granted the agency to decline entry? If concerns about the moral and ethical responsibilities of research and innovation become a matter of security, it is highly relevant to (re-)think carefully about the question to whom will science and innovation be responsible to (cf. Rychnovska 2016: 323).

Second, the case study has shown how the logics and rationales of RRI and security innovation are hard to reconcile when it comes to smart borders. Interview partners frequently referred to the struggles of applying RRI and its requirements of openness and engagement in the project while making sure that results are treated as confidential and information. Although for different reasons, industry actors in the project were at points unwilling to be transparent of the innovation process as required by the Horizon 2020 funding framework and their companies’ interests to protect innovation strategies and knowledge vis-à-vis competitors, again complicating the process of applying RRI in a meaningful way.

To conclude, this paper has pointed out how RRI represents by no means an apolitical space where social issues surrounding technology and innovation can be discussed and safeguarded against any political agenda. RRI itself embodies a political agenda embedded in a normative understanding of values that are considered ‘the right way to go’, in this case study about border management and more generally, the creation of a smart fortress Europe from the EU’s standpoint (cf. Djistelbloem and Meijer 2011). Consequently, the framework can be regarded as a political instrument for the governance of innovation and technology where the political state, in this case the EU, is central to the innovation process, rather than the publics.

The practical implementation of RRI has experienced a variety of well-known challenges, also in other fields, including how to deal with the diversity of societal values, finding the right balance between academic freedom and steering, and addressing uncertainty inherent in research and innovation (cf. Ulnicane 2020: 8). Yet, the central normative propositions of inclusiveness openness and interaction between science, policy and public that RRI promotes of ‘‘are inherently political discussions, involving considerations of power, democracy, and equity, and suggest that responsible innovation cannot, and should not, be decoupled from its political and economic context’ (Owen et al. 2013: 37). Applying the framework´s principles to the field of (border) security then lays bare the limitations of RRI for a field that might be more in need than any other to consider the ethical dimension of its activities. This paper has pointed only at some of these conflicts—that RRI itself embodies a political agenda embedded in a normative understanding of which values are considered the right ones, conceals alternative experiences by those on whom security is enacted upon and proposes openness and transparency throughout the research process that can hardly be met in practice due to confidentiality agreements. Thus, the application of RRI in the context of border security provides much more complex challenges which lay beyond both, the scope of this paper and the project under study but are highly relevant for future work in this field, both academic and policy-oriented. Future research would thus benefit from attending to these concerns and contextualizing RRI and its propositions for the specific field of security. It is precisely at the intersection of innovation security, responsibility where a much more profound reconsideration of the framework’s usefulness is needed—if it is supposed to be more than a comfortable add-on to European security research and governance.

## Footnotes

1 Dual-use knowledge refers to knowledge that can be utilized for military as well as civilian purposes (Witjes and Olbrich 2017).

2 The acronym stands for ‘BOrdDErGuArd’. The full project title is “Proactive Enhancement of Human Performance in Border Control”.

3 Grant agreement number 653676.
